# Play to Become a Surgeon: Impact of Nintendo WII Training on Laparoscopic Skills

**DOI:** 10.1371/journal.pone.0057372

**Published:** 2013-02-27

**Authors:** Domenico Giannotti, Gregorio Patrizi, Giorgio Di Rocco, Anna Rita Vestri, Camilla Proietti Semproni, Leslie Fiengo, Stefano Pontone, Giorgio Palazzini, Adriano Redler

**Affiliations:** 1 Department of Surgical Sciences, Faculty of Medicine and Dentistry; Sapienza University of Rome, Rome, Italy; 2 Department of Public Health and Infectious Disease, Faculty of Pharmacy and Medicine, Sapienza University of Rome, Rome, Italy; 3 Department of Radiological Sciences, Oncology and Pathology, Faculty of Medicine and Dentistry, “Sapienza” University of Rome, Rome, Italy; University of Colorado, United States of America

## Abstract

**Background:**

Video-games have become an integral part of the new multimedia culture. Several studies assessed video-gaming enhancement of spatial attention and eye-hand coordination. Considering the technical difficulty of laparoscopic procedures, legal issues and time limitations, the validation of appropriate training even outside of the operating rooms is ongoing. We investigated the influence of a four-week structured Nintendo® Wii™ training on laparoscopic skills by analyzing performance metrics with a validated simulator (Lap Mentor™, Simbionix™).

**Methodology/Principal Findings:**

We performed a prospective randomized study on 42 post-graduate I–II year residents in General, Vascular and Endoscopic Surgery. All participants were tested on a validated laparoscopic simulator and then randomized to group 1 (Controls, no training with the Nintendo® Wii™), and group 2 (training with the Nintendo® Wii™) with 21 subjects in each group, according to a computer-generated list. After four weeks, all residents underwent a testing session on the laparoscopic simulator of the same tasks as in the first session. All 42 subjects in both groups improved significantly from session 1 to session 2. Compared to controls, the Wii group showed a significant improvement in performance (p<0.05) for 13 of the 16 considered performance metrics.

**Conclusions/Significance:**

The Nintendo® Wii™ might be helpful, inexpensive and entertaining part of the training of young laparoscopists, in addition to a standard surgical education based on simulators and the operating room.

## Introduction

Over the past two decades video-games have become an integral part of the new multimedia culture. Their social and cultural significance has been widely investigated and they seem to be the basis of the so called “media competency” acquisition [1]. Today's video-game players include millions of individuals of all ages and backgrounds. In 2010, 72% of American households played games thus fueling a multi-billion dollar industry, spending $25.1 billion on video-games, hardware and accessories with $5.9 billion in revenue [2].

Despite some negative effects of excessive game playing [3], [4], several studies have confirmed that video-gaming enhances spatial attention and eye-hand coordination [5], [6].

For many people videogames are the first active approach to a bidimensional interface and they could increase familiarity with the screen interfaces used in laparoscopic surgery [6]. Compared to open surgery, laparoscopy presents different difficulties such as limited motion range of instruments, loss of depth perception, haptic feedback and fulcrum effect [7], [8]. Considering the technical difficulty of laparoscopic procedures, legal issues and time limitation, validation of appropriate trainings even outside of the operating rooms is ongoing [9], [10], [11], [12].

Several studies have investigated the relationship between video-gaming and surgical skills by analyzing the impact of prior gaming experience on surgical ability [13] and the influence of a systematic video-game training on laparoscopic simulator performance [14]. Data from the literature suggests that 3D game training is more beneficial than 2D [15].

Kolga Schlickum et al. compared a first-person 3D shooter game (Half Life, 1998, Sierra On-line, Los Angeles, California) with a bidimensional video-game (Chessmaster, 2004, Ubisoft, Montreuil-sous-Bois, France) showing that tridimensional games provide a greater transfer of training effect on virtual reality endoscopic simulators [14]. Half life was chosen for the similarities with virtual endoscopy: in fact navigation capabilities are mandatory in both applications and the authors concluded that transfer effect increases when increasing visual similarity. We wondered if a similar transfer effect could also be found in laparoscopic surgery, focusing our attention on Nintendo® Wii™.

Since a limited number of studies were conducted in this regard and with a very short training time, we investigated the influence of a four week structured Nintendo® Wii™ training on laparoscopic performance, by analyzing metric effects with a validated laparoscopic simulator (Lap Mentor™, Simbionix™).

## Materials and Methods

The study was performed in the Department of Surgical Sciences at “Sapienza” - University of Rome in Italy.

The study was approved by the local Ethics Committee (Protocol number 808/12). All participants were enrolled on a voluntary basis and each resident gave informed consent before enrollment.

Our research was structured in two sessions of tests on a laparoscopic simulator. Between these two sessions, participants were randomized into two groups. One group underwent a structured training with the Nintendo Wii console, while the other was asked not to play any video game. The re-test session was scheduled 28 days after session 1.

61 post-graduate I–II year residents in General, Vascular and Endoscopic Surgery were assessed for eligibility. Before entering the study, all participants completed a questionnaire assessing demographics, number of previous laparoscopic procedures (camera, assistant, operator) and average number of hours per week spent playing video-games.

Inclusion criteria were lack of prior laparoscopic simulator experience, none or low experience in laparoscopic surgery (less than 5 laparoscopic procedures, all of them as camera operator), low video-game experience (less than 1 hour a week in the last 10 years).

17 residents were excluded from the study because they did not met inclusion criteria and two residents declined to participate.

A total of 42 residents were then enrolled as the study subjects. Participants demographics, previous exposure to laparoscopy, exposure to laparoscopy during the study period and video games experience were reported in Table 1. Participants took part in laparoscopic procedures only as camera operators.

**Table 1:** Characteristics of study participants.

Characteristic	Group Control, n = 21	Wii, n = 21	p-value
Age (years)	27.6±1.5*	26.9±1.3*	0.121
Sex (female/male)	13/8	11/10	0.756**
Right handed	21	19	0.488
Previous laparoscopic procedures	2.2±1.2* Range 0;4	1.9±1.1* Range 0;4	0.311
Laparoscopic procedures during the study period	0.3±0.5* Range 0;1	0.2±0.4* Range 0;1	0.474
Residents in General surgery	11(44.0)	14(56.0)	0.688**
Residents in Endoscopic surgery	6(60.0)	4(40.0)	
Residents in Vascular surgery	4(57.1)	3(42.9)	
I year residents	12(44.4)	15(55.6)	0.260**
II year residents	9(60.0)	6(40.0)	

* mean ±sd;

** Pearson's chi-squared test; in brackets: percentage.

All participants were tested in the first session on our validated laparoscopic simulator (Lap Mentor™, Simbionix™) and then randomized to group 1 (Controls, no training with the Nintendo® Wii™), and group 2 (training with the Nintendo® Wii™) with 21 subjects in each group, according to a computer-generated list.

After four weeks all residents underwent a second testing session on the laparoscopic simulator with the same tasks evaluated as in session 1.

Between the two sessions, subjects in group 1 (control) were instructed not to play video-games while group 2 underwent a systematic Nintendo® Wii™ training for 60 minutes a day, five days a week, for four weeks. Simulator testing and Nintendo Wii training were conducted under supervision in a dedicated room of our Department with two Nintendo Wii consoles and one laparoscopic simulator station.

### Simulator testing

All participants were tested in three basic laparoscopic skills tasks and one virtual patient case of complete cholecystectomy (Lap Chole – Full Procedure Module number 1) on a validated laparoscopic simulator (Lap Mentor™, Simbionix™).

For each task we selected specific parameters calculated and reported by the simulator software to evaluate the subject's performance.

Before performing the first three tasks each participant viewed a standardized screen, provided by the simulator, illustrating the procedure.

In task 1 (0° camera manipulation), subjects must locate 10 balls and snap their photos using a 0° camera. We recorded the total time in seconds to complete the procedure and the accuracy rate in percentage calculated by dividing the number of correct hits by the total number of camera shots.
Task 2 required eye-hand coordination: participants must locate 10 flashing blue and red balls and touch them with the tool of the same color. We recorded total time, accuracy rate calculated by dividing the number of correct hits by the total number of touched balls and economy of movement of right (EMRI) and left instrument (EMLI), measured in percentage, calculated by dividing the ideal path length by the relevant path length of right/left instrument.
In task 3, designed for two handed maneuvers, participants had to expose with two grasping tools 9 balls hidden under a jelly mass and place them into an endobag. Total time, accuracy-rate, number of exposed green balls collected and economy of movement of right (EMRI) and left instrument (EMLI) were recorded.
In task 4 participants performed a complete virtual cholecystectomy after a standardized oral instruction on the surgical technique and a description of the patient's medical history reported by the simulator. All subjects selected procedure number 1 of Full Procedures LapChole. We evaluated the total time to complete virtual cholecystectomy, the efficiency of cautery (in percentage) calculated by dividing the time of cautery applied with appropriate contact with adhesions by the total cautery time, the safe cautery (in percentage) calculated by dividing the time of cautery applied more than 5 mm from the biliary system and the time of cautery applied more than 15 mm from the clip by the total cautery time and finally the number of movements of right (NMRI) and left instrument (NMLI).

### Nintendo® Wii™ training

The Nintendo® Wii™ (Nintendo Co. Ltd, 2006, Kyoto, Japan) is a video-game console with a wireless controller, the Wii™ Remote, which is a handheld pointing device, able to detect movement in three dimensions. Thanks to this controller, the gamers can play using physical gestures while traditional video-games require the player to press a button or to move a joystick.

We chose three games (Wii™ Sports Tennis, Wii™ Table Tennis and Battle at high altitude) with high demands of eye-hand coordination, movement precision, depth perception and 3D visualization, for the purpose of investigating the transfer effect of video-games on laparoscopic simulator performance.

In Wii™ Sports Tennis (Wii™ Sports), the Wii™ Remote is swung like a racket and the player's shots are determined by the controller direction. This game requires prolonged visual concentration and eye-hand coordination developing visual acuity and focus flexibility.
In Wii™ Table Tennis (Wii™ Sports Resort) there is a closer action field with less dynamic acuity but the player has greater control adding spin to the ball by twisting the Wii™ Remote.
Battle at high altitude is set in an archipelago: the player moves his aircraft with 20 balloons attached to the tail and the goal is to stay with as many balloons as possible within 3 minutes or to burst the opponent's balloons. This game requires precision of movements and an excellent 3D visualization rather than other skills.

All 21 participants belonging to the Wii Group underwent a systematic Nintendo® Wii™ training, using both hands, for 60 minutes a day, five days a week, for four weeks.

### Statistical analysis

Continuous variables are presented as mean ± SD. We assessed the normality of the data with Shapiro-Wilk test. Data of performance metrics do not follow a normal distribution and therefore were reported as median and interquartile range. Categorical variables are presented as counts or percentage. To evaluate the homogeneity and the differences between groups, we used the Mann-Whitney test; to evaluate the differences within groups we used the Wilcoxon test. We calculated, for all variables, the ratio between session 2 and session 1 (index numbers), to assess their modification from the initial test, expressed as percentage of improvement. The probability values are 2-sided; a probability value of less than .05 was considered statistically significant. All analyses were carried out with SPSS software version 18.0 (SPSS Inc., Chicago, Illinois).

## Results

No significant differences were found between the Wii and control group in session 1, in keeping with the homogeneity of the two groups for all the performance metrics evaluated. All 42 subjects improved significantly from session 1 to session 2 (table 2).

**Table 2:** Descriptive statistics of all variables of the two groups (session 1 and 2).

		Control Group		Wii Group		
	Performance metric	Median	IQR	Median	IQR	P-value
Task 1 (Session1)	Total time (s)	185.0	(142.0–207.5)	186.0	(131.5–209.5)	0.940
	Accuracy rate (%)	30.5	(22.4–35.7)	32.3	(21.6–36.8)	0.850
Task 1 (Session 2)	Total time (s)	166.0	(137.0–186.0)	136.0	(116.5–160.0)	0.021
	Accuracy rate (%)	32.0	(25.0–37.2)	52.4	(41.6–66.7)	<0.0001
Task 2 (Session 1)	Total time (s)	65.0	(54.0–72.5)	65.0	(56.0–73.5)	0.850
	Accuracy rate (%)	57.2	(54.8–61.8)	58.1	(53.1–65.2)	0.801
	EMRI (%)	37.1	(34.6–40.4)	37.8	(33.2–41.3)	0.801
	EMLI (%)	40.5	(38.4–44.2)	41.7	(36.7–44.9)	0.801
Task 2 (Session 2)	Total time (s)	54.0	(49.0–68.0)	52.0	(46.5–59.5)	0.170
	Accuracy rate (%)	61.0	(57.1–67.1)	82.8	(74.5–87.6)	<0.0001
	EMRI (%)	40.1	(37.1–44.1)	55.7	(51.3–65.7)	<0.0001
	EMLI (%)	44.0	(40.1–50.4)	62.8	(53.5–69.7)	<0.0001
Task 3 (Session 1)	Total time (s)	147.0	(103.5–168.0)	142.0	(110.0–159.0)	0.890
	Balls collected (n)	8.0	(7.0–8.0)	8.0	(7.0–8.0)	0.659
	EMRI (%)	22.5	(19.1–27.1)	22.6	(18.8–28.1)	0.940
	EMLI (%)	24.9	(21.2–30.0)	24.1	(19.8–31.4)	0.910
Task 3 (Session 2)	Total time (s)	111.0	(97.0–149.5)	100.0	(89.5–121.5)	0.159
	Balls collected (n)	8.0	(8.0–8.0)	8.0	(8.0–9.0)	0.084
	EMRI (%)	28.3	(22.7–33.5)	41.3	(32.9–50.7)	<0.0001
	EMLI (%)	29.4	(25.2–35.4)	47.2	(38.2–56.5)	<0.0001
Task 4 (Session 1)	Total time (s)	701.0	(647.0–784.5)	699.0	(622.5–797.0)	0.753
	Efficient cautery (%)	41.7	(38.4–45.2)	42.3	(38.5–45.4)	1.000
	Safe cautery (%)	61.5	(47.5–65.0)	58.4	(47.3–63.9)	0.678
	Perforations (n)	3.0	(2.5–4.0)	3.0	(2.0–3.5)	0.282
	NMRI (n)	511.0	(445.5–547.5)	489.0	(462.5–559.0)	0.860
	NMLI (n)	251.0	(200.0–361.0)	254.0	(202.0–356.0)	0.970
Task 4 (Session 2)	Total time (s)	655.0	(581.5–739.5)	565.0	(509.5–679.0)	0.027
	Efficient cautery (%)	45.8	(42.2–51.1)	58.8	(54.9–63.6)	<0.0001
	Safe cautery (%)	64.2	(52.2–69.5)	70.4	(64.6–78.6)	0.014
	Perforations (n)	2.0	(2.0–3.0)	1.0	(1.0–2.0)	0.001
	NMRI (n)	456.0	(400.0–515.5)	371.0	(329.5–417.0)	0.006
	NMLI (n)	240.0	(178.5–300.0)	188.0	(158.0–244.5)	0.024

IQR: Interquartile range; EMRI: economy of movement of right instrument; EMLI: economy of movement of left instrument;

NMRI: number of movements of right instrument; NMLI: number of movements of left instrument.

The control group improved significantly in all performance metrics except for:

The accuracy rate measured in task 1 (0° camera manipulation) and the total time to complete it (p values respectively of 0.144 and 0.092).
The total number of exposed balls collected in task 3 (two handed maneuvers), p = 0.470.

The Wii group improved significantly in all performance metrics (table 2).

Comparing the Wii with the control group we found significant differences in the improvement for all the performance metrics measured (p<0.05) except in three cases (Table 2):

The total time needed to complete task 2 (eye-hand coordination), p = 0.170.
The total time needed to complete task 3 (two handed maneuvers), p = 0.159.
The total number of exposed balls collected in task 3, p = 0.084

**Figure 1:**
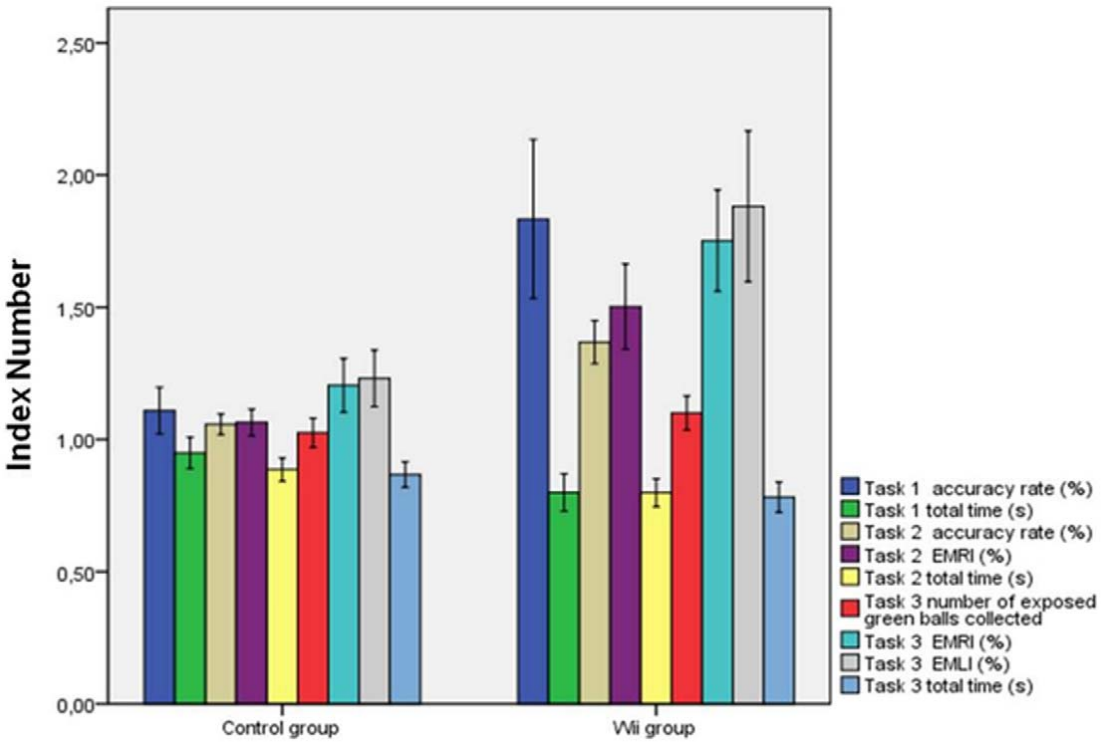
Comparison of the results for laparoscopic basic skills of the two groups (index numbers).

**Figure 2:**
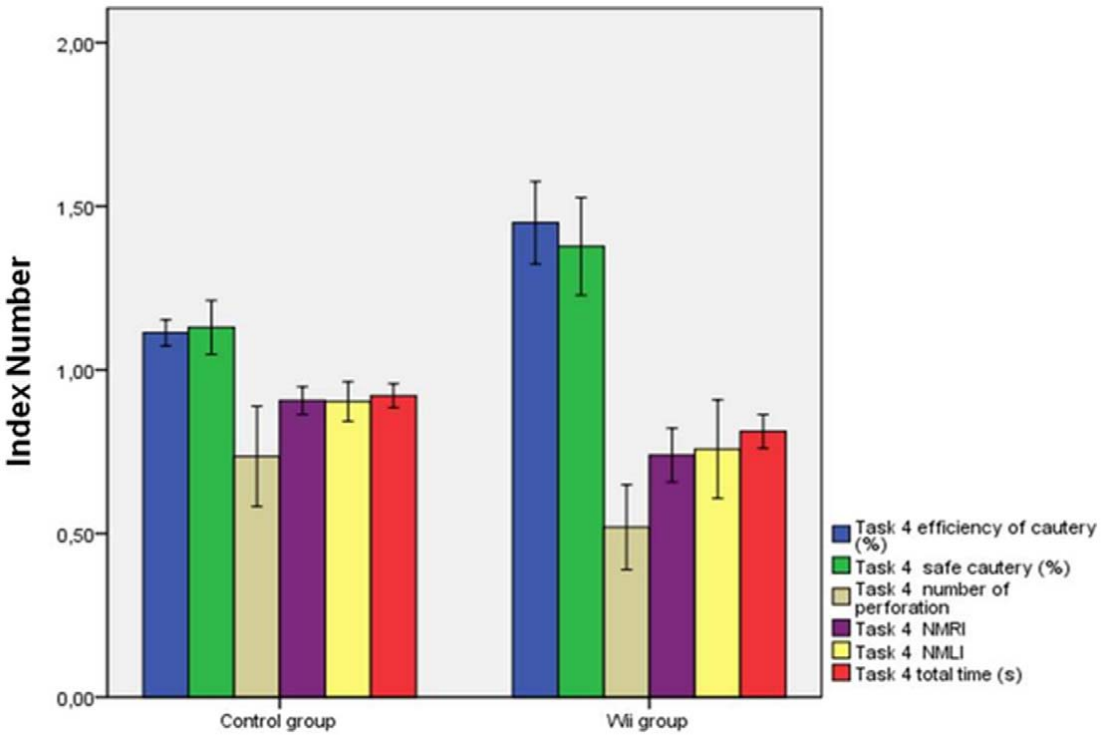
Comparison of the results for virtual full cholecystectomy of the two groups (index numbers).

Analyzing the variables as the ratio (index numbers) we can express the improvements, between the two groups, in percentage terms as shown in Table 3, Figures 1 and 2.

**Table 3:** Percentage of improvement for Wii and Control Group between session 1 and session 2.

		Control Group	Wii Group
	Performance Metric	Improvement (%)	Improvement (%)
Task 1	Total time	6	21
	Accuracy rate	10	83
Task 2	Total time	12	21
	Accuracy rate	5	36
	EMRI	6	50
	EMLI	10	51
Task 3	Total time	14	22
	Balls collected	2	10
	EMRI	20	75
	EMLI	23	88
Task 4	Total time	8	18
	Efficient cautery	11	42
	Safe cautery	12	35
	Perforations	27	49
	NMRI	10	24
	NMLI	10	22

EMRI: economy of movement of right instrument; EMLI: economy of movement of left instrument;

NMRI: number of movements of right instrument; NMLI: number of movements of left instrument.

## Discussion

Several studies have postulated a correlation between video-game experience and surgical skills. Probably in both areas the same process of “perceptual learning” is involved. Video-game players and surgical trainers improve their performance on subsequent attempts using an attentional weighting: they focus more attention on important aspects discarding the irrelevant [16]. This correlation is of more than theoretical importance and opens an important debate in terms of surgical education.

In recent years, technical difficulties of laparoscopic procedures, legal issues and time limitations have increased the need to perform training even outside of the operating theater [9], [10], [11], [12]. Laparoscopic simulators represent a satisfactory response to this request but their high costs have limited their spread. Video-games may be a cheap and widely available product, helping to develop cognitive skills that, apparently, can be transferred in improved surgical performance.

Rosser et al. tested 33 surgeons with three different video-games and a laparoscopic simulator, showing increased simulator performance in subjects with prior and current video-games experience [6]. Rosemberg et al. evaluated 11 medical students playing a selection of video-games and performing laparoscopic tasks in a swine model. As reported in other studies they found a correlation between video-game play and the completion time of simple laparoscopic tasks, such as object translocation, while no advantages were identified in more complex tasks [17]. Schlickum et al. were the first to demonstrate the influence of a five-week systematic video-game training on endoscopic simulator performance even in complex tasks. They used a 3D shooter game (Half Life) chosen for the similarities with virtual endoscopy [14]. The study demonstrated that transfer effect increases when increasing visual similarity and this concept is central even in our research.

For the training-period, we used a Nintendo® Wii™ console which could reproduce the movements of laparoscopy more closely than conventional 2D or 3D video-games due to its exclusive technology based on a motion-sensing interface. As in laparoscopic surgery, the action is transferred to a 2D representation on a monitor creating a 3D reconstruction in the player/surgeon's mind. Badurdeen et al. investigated the correlation between Nintendo® Wii™ skills and laparoscopic skills tested with three tasks on a webcam based laparoscopic simulator on 20 participants. They concluded that medical students and junior doctors with major Nintendo® Wii™ ability perform significantly better at laparoscopic tasks [18]. Although this research has shown a link between Wii™ skills and laparoscopic performance, only a further study conducted by Boyle et al. investigated whether training on Nintendo® console can improve laparoscopic ability on a ProMIS™ surgical simulator. However, the study was conducted with a rather small number of participants (22 medical students) and with a very short training period (3 hours), showing a significant improvement in only one of the performance metrics evaluated [19].

Our study has provided a four week structured Nintendo® Wii™ training, focusing on games with high demand for visual concentration and eye-hand coordination. We decided to stress this latter aspect based on a recent study conducted by Wilson et al. on perceptual impairment and psychomotor control in virtual laparoscopic surgery [20].

Gaze analyses has shown that expert laparoscopic surgeons make fewer movements and spend more time fixating the target than novices, who divided the observation time between the targets and tracking the tools. We therefore hypothesized that training with games that stimulate target focusing could improve gaze strategy and economy of movement even in laparoscopic tasks. In concordance with this hypothesis, we observed a significant reduction of movements, altogether with an increased economy of the path of instruments and accuracy rate. The first problem that surgeons in training have to face in laparoscopy is the transposition of 3D movements in a 2D view. This problem can be reduced in different ways, including laparoscopic simulators and video-games. For this purpose, video-game consoles detecting movement in three dimension and using physical gestures have proven to be more efficient than traditional video-games with joysticks. In our study, the Nintendo® Wii™ proved its validity in improving both the basic laparoscopic skills and complete simulated procedures of cholecystectomy.

In analyzing our results, even though a reduction of time was observed between sessions 1 and 2, the time to perform certain exercises did not change significantly between the two groups. In fact, training on the Wii™ affects, in a highly significant manner, the accuracy and economy of movement of the instruments. In full procedures, the training reduced the complication rate and the unsafe cautery rate, which are probably the most frequent avoidable incidents for novice laparoscopists. This can be translated in surgery with the acquisition of more precise and finalized movements. Therefore a structured training on laparoscopic simulators in association with the Nintendo® Wii™ video-games might help optimizing the learning-curve in laparoscopy.

Our study must be interpreted in the context of some limitations. First the group of residents enrolled was heterogeneous for surgical experience even if homogeneously distributed in both groups (Table 1).

As this is a pilot study of feasibility for such tests to provide preliminary evidence on the efficacy of different educational training interventions, one of the limits is the relatively short training session on the video-game console. It would be very interesting to extend the Wii-training period in order to verify the effect even in later stages of the learning curve. We wish in further studies to widen the training to minimize the familiarization phenomenon that typically affects the first approaches to a new device [21], [22].

The familiarization can be observed in any experiment when more than one session is scheduled on the same instrument: the first time, subjects get to know the device and the second (or third) time, they have already been in contact with it and they are consequently more familiar to it. This is probably the main reason why, between session 1 and 2, the control group improved significantly in almost all performance metrics (table 2). The same effect can be observed in the Wii Group but the addition of training on video games explains why improvement were more evident and significant. Therefore we should preliminarily evaluate when the plateau of the learning curve is reached in our tests and see if 3D video game training might have an influence at that time. The optimum would be to develop a dedicated software to run on the console stressing two-hand coordination, depth perception and precision of movements, before studying how such training can be transferred in real surgical procedures in the operating room.

## Conclusions

It is hard to suggest that Academic Institutions adopt a video-game console as a didactic tool for surgery in addition to traditional training and simulators, even though our study proved its efficacy in improving the skills of surgeons in training both for laparoscopic basic skills and full virtual cholecystectomy procedures. We hope this may be a trigger to develop dedicated software aimed to help young surgeons as the economic impact of these consoles is significantly lower than traditional laparoscopic simulators and they provide a basic didactic value. The Nintendo® Wii™ may be adopted in lower-budget Institutions or at home by younger surgeons to optimize their training on simulators before performing real procedures.
